# Oxidized Extracellular DNA as a Stress Signal in Human Cells

**DOI:** 10.1155/2013/649747

**Published:** 2013-03-06

**Authors:** Aleksei V. Ermakov, Marina S. Konkova, Svetlana V. Kostyuk, Vera L. Izevskaya, Ancha Baranova, Natalya N. Veiko

**Affiliations:** ^1^Research Centre for Medical Genetics, Russian Academy of Medical Sciences, Mosskvorechie street 1, Moscow 115478, Russia; ^2^Center for the Study of Chronic Metabolic Diseases, School of System Biology, George Mason University, Fairfax, VA 22030, USA

## Abstract

The term “cell-free DNA” (cfDNA) was recently coined for DNA fragments from plasma/serum, while DNA present in *in vitro* cell culture media is known as extracellular DNA (ecDNA). Under oxidative stress conditions, the levels of oxidative modification of cellular DNA and the rate of cell death increase. Dying cells release their damaged DNA, thus, contributing oxidized DNA fragments to the pool of cfDNA/ecDNA. Oxidized cell-free DNA could serve as a stress signal that promotes irradiation-induced bystander effect. Evidence points to TLR9 as a possible candidate for oxidized DNA sensor. An exposure to oxidized ecDNA stimulates a synthesis of reactive oxygen species (ROS) that evokes an adaptive response that includes transposition of the homologous loci within the nucleus, polymerization and the formation of the stress fibers of the actin, as well as activation of the ribosomal gene expression, and nuclear translocation of NF-E2 related factor-2 (NRF2) that, in turn, mediates induction of phase II detoxifying and antioxidant enzymes. In conclusion, the oxidized DNA is a stress signal released in response to oxidative stress in the cultured cells and, possibly, in the human body; in particular, it might contribute to systemic abscopal effects of localized irradiation treatments.

## 1. Introduction

The effect of information transfer from the irradiated cells (target cells) to adjacent, nonirradiated ones is known as the bystander effect (BE). The BE was shown for a number of damaging agents of both physical and chemical nature, in many types of eukaryotic cells, and covers a variety of physiological effects including the genomic instability, the cell death, and/or the adaptive response (AR) [1]. As a result of adaptive response brought about by low-dose ionizing radiation, the cells develop resistance to further irradiation at higher (damaging) doses. Both reactions (AR and BE) are closely interconnected biologically and have many similarities and characteristic features [2–5]. Interestingly, both AR and BE may be transferred to intact cells through their exposure to the media conditioned by exposed cells [6, 7]. Importantly, the development of particular variant of cellular response depends on the amount of irradiation, amount of cells, their tissue origin, and the stage of the cell cycle. In some experimental studies, the response of bystander cells might not be adaptive [1–7].

For the first time, the intercellular signaling was experimentally demonstrated on Chinese hamster cell culture [8]. Following irradiation of not more than 1% of cellular nuclei, the authors observed increased frequency of sister chromatids exchanges in 20–40% of the cultured cells. It is generally accepted that there are three possible pathways of signal transfer from the irradiated cell to the “bystander” cell: through the direct cellular contact with the formation of common membranous structures, through interaction involving the gap junctions or via the signals released to the culture medium of the irradiated cells. The third pathway is typical for the BE induced by radiation with low Linear Energy Transfer (LET) [9]. Many candidate molecules, mainly the soluble proteins, have been proposed as mediators of the bystander signaling between treated cells and bystander cells. All these data had been reviewed in details previously [10–17].

In course of our studies, we thoroughly evaluated an idea of existence of certain intrinsic cellular factor that is released from the dying cells, thus, causing the development of the bystander effect. The present work is a brief overview of our recent findings concerning the possible role of extracellular DNA oxidation in the development of the adaptive response and bystander effect, as triggered in human cells by exposure to oxidative stress [18–35].

## 2. Oxidative Stress Induces the Oxidation of Cellular DNA

Many chronic diseases are accompanied by an increase in overall oxidation of genomic DNA. Under oxidative stress, the DNA bases are prone to oxidation, with the most common products being the thymidine glycol and 8-hydroxy-2′-deoxyguanosine (8-oxodG). In fact, the 8-oxodG is the most widely used “marker” for oxidative DNA damage. The 8-oxodG is formed in DNA either via direct oxidation or can be incorporated into DNA by DNA polymerase as a modified base drawn from the nucleotide pool [36, 37].

Previously published studies have reported the frequency of 8-oxodG in genomic DNA (gDNA) samples. For example, gDNA extracted from cultured cells [38, 39] contains approximately from 0.1 to 0.5 8-oxodG per 10^6^ nucleotides, while normal breast tissue from cancer patients has significantly higher levels of oxidative DNA damage—up to 25 8-oxodG per 10^6^ nucleotides [40]. Most of the results clearly indicate higher steady-state levels of modified DNA bases in cancerous tissues than in their cancer-free surrounding tissues. The level of oxidative modification of cellular DNA may serve as a predictive marker of cancer development [41–43]. For example, in breast carcinomas, 8-oxodG levels have been reported as being 8 to 17 times higher as compared with nonmalignant breast tissue [44–46]. Additionally, it was shown that an exposure of the MCF-10A cells to doxorubicin leads to a significant increase in the levels of eleven different oxidized forms of DNA bases [47].

The genomes of prostatic carcinoma cell lines LNCaP, DU145, and PC3 contain between 3 and 4.5 8-oxodG/10^6^ nucleotides, while genomic DNA extracted from the prostatic tissue of young men contains approximately *∼*50 8-oxodG/10^6^ nucleotides [48, 49]. With age, these levels increase to up to *∼*75 8-oxodG/10^6^ nucleotides. Noncancerous prostatic tissue of prostatic carcinoma patients contains *∼*90 8-oxodG/10^6^ nucleotides, while in prostatic carcinoma cells these amounts increase up to *∼*120 8-oxodG/10^6^ nucleotides [48, 49]. Interestingly, in prostatic carcinoma cells, the levels of 8-oxodG could be induced by exposure to supraphysiological concentrations of dihydrotestosterone [50].

Human ovarian tissue contains approximately 1.3 8-oxodG per 10^6^ nucleotides, while in advanced epithelial ovarian carcinoma these levels increase to 2.2 8-oxodG/10^6^ nucleotides [51]. In uterine myomas, the levels of 8-oxodG are higher than those in underlying myometrium (*∼*3-4 8-oxodG and 2 8-oxodG/10^6^ nucleotides, resp.) and correlate with the size of the tumor [52]. 

Two-three-fold increases in levels of 8-oxodG were observed in lung carcinoma as compared to cancer-free surrounding lung tissues [53]. Among Noncancerous lung samples, the lung tissue removed from smokers had the highest increases of modified bases above the control levels and the highest overall amounts of 8-oxodG [54].

DNA extracted from PBMCs of healthy donors contains between 1 and 1.3 8-oxodG bases per 10^6^ nucleotides [55–60]. In PBMCs of cancer patients, the content of 8-oxodG increases to 1.5–1.8 modified bases per 10^6^ nucleotides [45, 55, 56, 59, 60]. Lymphocyte DNA from lung cancer patients had 1.7-fold higher levels of 8-oxodG compared to the controls. The difference was especially evident in current smokers [61]. The levels of 8-oxodG in DNA extracted from leukocytes of patients with Leber's hereditary optic neuropathy were 3.3 bases per 10^6^ nucleotides [62]. Similar increases were observed in DNA of Parkinson's disease and multiple sclerosis patients [63, 64]. Increased levels of 8-hydroxyadenine, 8-oxodG, thymine glycol, Fapy-guanine, 5-hydroxymethyl-2′-deoxyuridine, and Fapy-adenine were observed in brains of patients with Alzheimer's disease [65]. The mean levels of 8-oxodG in neurons were 10 times higher in elderly “poor-outcome” schizophrenia than in control DNA samples [66]. A marked elevation of 8-oxodG in leukocyte DNA samples obtained from patients with chronic renal failure [67–69] and Fanconi anaemia [70] was also reported. In germ-line cells of men with type 1 diabetes, the content of 8-oxodG is increased up to 9 modified bases per 10^6^ [71]. In patients with coronary artery disease, the levels of oxidative DNA damage correlate with the severity of the disease [72, 73]. Moreover, both in rats and in humans, the 8-oxodG content in DNA positively correlates with the age [74–79]. 

In the majority of studies, the levels of 8-oxodG were experimentally measured in total DNA extracted from the lysed cells or tissue samples. However, it is likely that some DNA molecules within the cell are substantially more prone to oxidative damage, for example, the mitochondrial DNA. As compared to the genomic DNA, the mtDNA shows substantial enrichment in GC nucleotide pairs; therefore, oxidized mtDNA may be disproportionate contributor to evaluated levels of 8-oxodG content in total DNA samples [80–82]. It should be noted that GC-rich fragment within genomic DNA tends to accumulate oxidative damage as well. One example of the preferentially oxidized DNA locus is the transcribed area of the ribosomal repeats [83]. 

All of this indicates that oxidation of DNA takes place in human cells as it is commonly observed both in health and disease. In cells undergoing oxidative stress and in chronic diseases, the levels of oxidative modifications of DNA increase substantially.

## 3. Extracellular DNA Is Enriched with the Oxidized Genomic DNA

The term “cell-free circulating DNA” (cfDNA) was coined for DNA fragments that could be collected from plasma, serum, or other bodily fluids. CfDNA circulates throughout the bloodstream of both healthy people and patients with various diseases. DNA isolated from cell-free supernatants of cells cultivated *in vitro* is known as extracellular DNA (ecDNA) [84]. EcDNA is found in the culture medium of both intact cells and cells exposed to various types of oxidative stress. 

The most widely accepted hypothesis is that the main sources of cfDNA/ecDNA are the dead cells [85]. Another hypothesis suggests that cfDNA/ecDNA could be actively excreted into the medium by living cells [86]. Recently, cfDNA got recognition as a promising biomarker for noninvasive diagnostics and monitoring of various diseases [84]. However, the biological role of cfDNA in normal or pathological conditions remains unclear. The functionality of these circulating DNA fragments is determined by cfDNA properties, for example, its concentration in the blood plasma and the level of oxidative modification that can be approximated by its average content of 8-oxodG. 

In plasma of healthy individuals, total cfDNA concentrations vary from 1 to *∼*100 ng/mL. These concentrations increase with age or in presence of various stressful conditions, for example, pregnancy, intensive exercise, or strong emotions as well as when malignancy or other chronic pathology is diagnosed. In plasma samples of patients with cancer or critical cardiovascular conditions, the concentrations of cfDNA increase up to 1000 ng/mL [84–92].

Oxidative stress is known to cause the DNA damage. The cells with the most damaged DNA die either by necrosis or by apoptosis. The oxidized DNA released from the dying cells is likely the most prominent contributor to cfDNA/ecDNA pool. Therefore, it is likely that cfDNA/ecDNA would contain larger amounts of 8-oxodG as compared to that in cellular DNA.

The comparative data describing 8-oxodG levels in cfDNA/ecDNA and in cellular DNA extracted from the same organism or cell culture are sparse. Some authors point that the levels of 8-oxodG in serum samples profiled by ELISA and by HPLC differ substantially [93, 94]. In our opinion, most likely explanation of these discrepancies is the fact that, in serum, 8-oxodG circulates both as free nucleoside and as part of oxidized cfDNA fragments. ELISA quantifies total concentrations of 8-oxodG that, in healthy donors, is around 0.3–5.9 ng/mL of plasma/serum [95–99] while HPLC-based techniques detect only free 8-oxodG that is present in plasma/serum samples at concentrations of 0.013–0.022 ng/mL [93, 94, 100–103]. One may calculate the minimal background content of 8-oxodG embedded in cfDNA fragments by subtracting bound 8-oxodG concentrations from total 8-oxodG concentrations (0.3–0.022 *≈* 0.28 ng/mL). Given that maximal observed concentration of cfDNA is at *∼*1000 ng/mL, the minimal content of 8-oxodG in cfDNA could be estimated as at least 280 8-oxodG bases per 10^6^ nucleotides, the figure that substantially exceeds estimated 8-oxodG content in DNA of living cells.

In our study we demonstrated that cfDNA/ecDNA is substantially enriched in 8-oxodG as compared to cellular DNA, up to 220–3000 8-oxodG per 10^6^ nucleotides. The degrees of enrichment were significant when cfDNA/ecDNA and cellular DNA samples were evaluated for cancer or myocardial infarction patients, for primary tumor cells, as well as for endotheliocytes cultures that were irradiated or treated with peroxide [30, 33]. 

In addition to preferential enrichment of cfDNA pools with oxidized DNA of dying cells, the contents of 8-oxodG in cfDNA may depend on well-known phenomenon of somewhat slowed down degradation of GC-rich DNA fragments in human serum as compared to AT-rich fragments [21, 92, 104–106]. Moreover, under the condition of oxidative stress, an increase in proportions of mitochondrial DNA within cfDNA was documented [80–82]. This process is relevant as mitochondrial DNA, on average, contains larger amounts of 8-oxodG as compared to genomic DNA [107].

## 4. Oxidized cfDNA/ecDNA Is a Stress Signal

The cfDNA extracted from blood plasma of patients with high oxidative stress levels can significantly influence the physiological activity of intact cells. For example, when primary endotheliocytes (HUVECs) were exposed to cfDNA samples obtained from patients with hypertension and atherosclerosis, their NO contents substantially decreased, while the DNA samples obtained from healthy donors have no effect of NO release [28, 29]. In electrically paced cultures of ventricular neonatal rat myocytes, an exposure to the cfDNA of patients with acute myocardial infarction has produced a decrease in the frequency of contraction [108]. The cfDNA from ischemic rats decreased the levels of ROS production in neuronal cultures [32]. Both ecDNA collected from the media of primary tumor cells cultures and cfDNA extracted from plasma of cancer patients have influenced ROS production in mesenchymal stem cells (MSCs) [33]. Importantly, cfDNAs extracted from blood of myocardial infarction and rheumatoid arthritis patients stimulate the expression of DNA sensor toll-like receptor 9 (TLR9) in MSCs, while an exposure to gDNA did not influence TLR9 levels [35].

As observed both in endothelial cells and in MSCs, the samples of the genomic DNA that were oxidized *in vitro* with either H_2_O_2_ or Methylene Blue (gDNA^OX^) evoke responses that are similar to those of cfDNA/ecDNA. In endothelial cells, exposure to gDNA^OX^ stimulated an expression of *NOX4* and suppresses *eNOS*, therefore, augmenting net production of ROS and decreasing the levels of NO [34]. In mesenchymal stem cells, increased concentrations of gDNA^OX^ and oxidized cfDNA/ecDNA stimulated a rapid increase in ROS synthesis and upregulated expression levels of the NF-E2-related factor-2 (NRF2), that plays a central role in antioxidant-response-element- (ARE-) mediated induction of phase II detoxifying and antioxidant enzymes along with a number of antioxidant response genes [33].

In murine macrophages, the treatment with GC-rich DNA fragments that are also enriched in 8-oxodG stimulates secretion of TNF-*α* [109]. The treatment of experimental animals with gDNA^OX^ produced inflammation and induced production of DNA^OX^-specific antibodies [110, 111]. 

An analysis of the data concerning cfDNA/ecDNA properties and the effects it produces on mammalian cells allowed us to suppose that ecDNA of irradiated cells (ecDNA^R^) may somehow influence the other nonirradiated cells within the cell cultures thus acting as a soluble stress-signalization factor in a radiation-induced BE. Our further studies confirmed this assumption, having for the first time demonstrated the significance of the bystander signaling with participation of oxidized extracellular DNA for human cells exposed to low-dose irradiation [22–27, 30, 31, 34].

## 5. Oxidized DNA-Dependent Signaling in Radiation-Induced Bystander Effect

### 5.1. EcDNA^R^ from the Irradiated Cells Is the Signaling Factor in BE

The main source of the ecDNA is the dead or dying cells. In a number of recent studies we demonstrated that ionizing low-LET irradiation increases the rate of apoptosis in various cell cultures. It seems that some subpopulations of cultured cells possess an increased sensitivity to apoptosis that may be evoked by irradiation at low doses. To pursue this hypothesis, we isolated and characterized the population of irradiation-sensitive human lymphocytes. This subpopulation was rich in large-size activated cells, could spontaneously incorporate (3H)-thymidine, had increased radiosensitivity, and decreased activity of the excision repair, as well as a high level of spontaneous chromosomal aberrations and apoptosis, all these increasing after irradiation [112]. 

In our study, the apoptosis levels were assessed by evaluating the number of double-strand breaks (DSBs) in the genomic DNA by using a technique based on visualization of phosphorylated protein H2AX (*γ*-foci) in the site of rupture. Accumulation of *γ*-foci in large amounts is indicative of the apoptosis [113]. Both in HUVECs and MSCs [26, 30], an irradiation is followed by accumulation of *γ*-foci. In peripheral blood lymphocytes, an irradiation leads to an increase in the activity of caspase-3, one of the main cysteine proteases activated in apoptosis [20, 22, 23]. After irradiation, the dying cells release the fragments of chromatin, thus, contributing to the pool of ecDNA/cfDNA.

The electrophoretic analysis shows that the size of ecDNA fragments produced by cultured cells varies from 180 to 20,000 bp, with a predominance of the fragments 180 and 360 bp in size that corresponds to mono- and dinucleosomes, respectively [20, 22, 30]. After irradiation, the concentration of longer fragments decreases and that of the short ones increases. EcDNA of irradiated cells contained significantly larger amounts of oxidation marker 8-oxodG than ecDNA of control (nonirradiated) cells or cellular DNA of irradiated cells [30].

The studies of the bystander effect were performed in various cell types, including G0-lymphocytes of peripheral blood [22–24, 27], HUVECs [30, 34], and MSCs of adipose tissue [26, 31]. Ionizing radiation is known to render both a direct effect on cellular structures via hitting with an energy quantum or particle and an indirect effect mediated by free radicals [114]. The cellular response to irradiation depends on many factors, but the most important of them is a substantial increase in the levels of ROS. Ionization results in synthesis of ROS. The process of ROS formation after exposure to radiation takes place within the time frame of several seconds to 2–5 minutes [115]. In turn, ROS induces multiple lesions in cellular DNA, including the ruptures of desoxyribose rings, the appearance of apurinic and apyrimidinic sites, single- and double-strand breaks, DNA protein cross-links, and formation of oxidized bases [116–122].

Importantly, in control (nonirradiated) cells, the ecDNA collected from the media conditioned by irradiated cells stimulates an increase on the ROS production to approximately the same degree as DNA oxidized *in vitro* or small doses of irradiation [34]. This indicates that ecDNA released from dying irradiated cells may serve as a stress signal that conveys a bystander effect, while ecDNA of nonirradiated cells is not a stress signal as it does not induce ROS synthesis in control cells.

Various parameters of the target cell and bystander cells are being analyzed in regards to irradiation and its effects. The most commonly studied group of such parameters includes a number of cytological characteristics of cellular nuclei, including the shape of the nucleus as well as FISH-defined descriptors of chromosomal territories, that is, positions of chromosome loci as they relate to the centre of the nucleus and to each other [18]. One of the known markers for irradiation-induced chromatin rearrangement is a position of pericentromeric loci of chromosome 1 (1q12). When effects of irradiation at a dose of 10cGy were compared to those of direct oxidative stress causing agent H_2_O_2_, of exposure to ecDNA extracted from the media conditioned by irradiated cells (ecDNA^R^) or of exposure to ecDNA extracted from H_2_O_2_-treated cells (ecDNA^H_2_O_2_^), similar structural rearrangements of chromatin were observed. Particularly, there was a decrease of the proportion of cells with the perimembranous location of loci at 1q12 and an increase in the proportion of the cells with central nuclear localization of these loci. It was also shown that exposure induces approximation of the loci 1q12 of homologous chromosomes 1 within the space of the cellular nucleus [20, 22, 23, 26, 27, 30, 31]. Additionally, the nuclei of both HUVECs and adipose-derived MSCs acquired a compacted, more spherical shape [26, 30, 31]. All these effects were primarily dependent on an increase in the production of ROS that was approximately to the same degree stimulated by irradiation, H_2_O_2_, ecDNA^R^, or ecDNA^H_2_O_2_^. When an inhibitor of ROS, *α*-tocopherol, was added to the media, all these effects were blocked.

Irradiation-dependent chromosomal loci relocation effects were confirmed by other researchers. The transposition of chromatin regions within the nucleus is accompanied by DNA repair [123]. Moreover, within the nuclei of irradiated cells, the convergence of homologous chromosomes in the sites of DSB emergency repair has been observed [124]. The structural rearrangement of chromatin promotes the launch of gene expression program that is necessary for the development of the adaptive response, with the approximation of chromosomes themselves being an event favorable for further elimination of DSBs through the repair associated with chromosomal homologous recombination (HR) or nonhomologous end joining (NHEJ). The irradiation-dependent chromosomal loci transposition had been demonstrated in lymphocytes [18–20, 22–24, 27], human endothelial cells [30], mesenchymal stem cells [26, 31], and in cancer stem cells of the mammary gland [25], thus, strongly suggesting that these effects are widespread. It should be mentioned that when irradiated cells fail to transpose the marker loci following irradiation, elevated levels of cell death are observed already at very low doses of ionizing radiation [25].

The structural transformations of chromatin mentioned above are accompanied by activation of ribosomal gene transcription which may be evidenced by staining the cellular preparations with silver nitrate or assessing rRNA levels by quantitative PCR. ROS-dependent induction of adaptive response implies an increase in the synthesis of proteins, primarily of repair proteins and those necessary for reorganization of the genome. Therefore, an enhanced transcription of ribosomal genes and an elevated amount of rRNA in ROS stimulated cell is to be expected [19, 23, 27, 30].

An increase in F-actin polymerization was observed both in irradiated cells and in ecDNAR-treated bystander cells. Alterations in the architecture of the cellular cytoskeleton observed after exposure to X-ray radiation and ecDNAR are similar as well [30]. Our findings suggest that alterations in the architectonics of the cellular cytoskeleton appear both after exposure to X-ray radiation and ecDNA^R^ [30].

The study also showed that addition of ecDNA^R^ into the growth medium of intact endotheliocytes leads to a decrease in the number of cells with single *γ*-foci and to a considerable increase in the number of apoptotic cells in the population [30]. Similar effects were observed when cells were irradiated in low doses. This study supports the findings of other authors having shown that an incubation medium of irradiated cells induces the initial stages of the apoptotic cascade in bystander cells. In these experiments, an induction of apoptosis in bystander cells was also accompanied by an elevation in the content of ROS within 6-hour time frame [125]. 

The mirror-like patterns of the effects described above and seen in both treated and bystander cells point to the transfer of a stimulus from irradiated to bystander cells. An addition of the ecDNA produced by control (nonirradiated) cells to the medium of bystander cells does not produce any of the effects described above, and no adaptive response is observed. Interestingly, after the hydrolysis by DNAse I, the ecDNA^R^ produced by irradiated cells loses its stress signal properties and its ability to evoke an adaptive response [20, 22].

It is also important to note that BE is not cell-type specific. The media conditioned by irradiated cells of one cellular type conveys the bystander effect to other kinds of bystander cells exposed to this media [24]. Similarly, ecDNA^R^ extracted from the growth medium of irradiated endotheliocytes conveys an adaptive response in the bystander MSCs and *vice versa* (data not published). 

### 5.2. EcDNA Signal Propagates with Aid of Oxidative Stress

The data described above indicate that the cascade of sequential events in ecDNA-signaling may be as follows:

Irradiation → [*primary oxidative stress* → oxidation of gDNA → apoptosis of some portion of irradiated cells → release of oxidized ecDNA^R^ → reception of the ecDNA^R^ signal by the bystander cells → *secondary oxidative stress*] → oxidation of gDNA in the bystander cells → apoptosis of some portion of bystander cells → release of oxidized ecDNA, and so forth.

**Figure 1:**
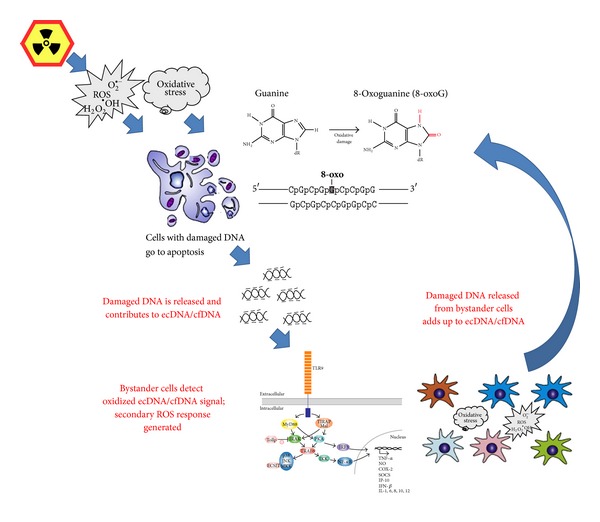
The proposed mechanisms for the propagation of the stress signal from irradiated cells to bystander cells. In this scheme, the 8-oxo-dG serves as a model example of DNA lesion that turns DNA fragments into the stress signal; it should be noted that other types of DNA lesions may be recognized as well. The central player that ensures amplification of the signal in this cascade is the oxidative stress. The secondary oxidative stress evoked in intact bystander cells occurs after an interaction of the oxidized ecDNA with the receptors, or oxidized DNA sensors, that must be present on the surface or inside the bystander cells. One possible candidate for oxidized DNA sensor is toll-like receptor TLR9.

In this cascade, the oxidative stress propagates from irradiated cells to bystander cells (Figure 1). The secondary oxidative stress that is evoked in intact bystander cells occurs after an interaction of the oxidized ecDNA^R^ with its receptors, or oxidized DNA sensors, that must be present on the surface or inside the bystander cells. The possible candidates for these sensors are the transmembrane proteins of the toll-like receptor family, namely, TLR9 [126]. Being transmembrane receptors, they contain a repetitive LRR domain capable of binding the ligand and a highly conservative intracellular region that ensures the interaction between the receptors and the molecules of the downstream signaling pathway, for example, an adapter protein MyD88. It is well known that the DNA fragments with unmethylated CpG motifs may serve as TLR9 ligands. In this cascade, the formation of the “DNA-TLR9” complex initiates the cellular signaling pathway that, in turn, leads to an activation of the transcription factor NF-*κ*B, which in many different ways augments the biosynthesis of ROS. For example, TLR9 ligation may be followed by an increase in intranuclear production of NO^•^ [127, 128] or O_2_^−^ radical [129]. In human monocytes, the binding of CpG-DNA to TLR9 is accompanied by secretion of both NO^•^ and ROS [130], while in neutrophils it leads to the production of peroxynitrite [127]. The slow-acting oxidants O_2_^•−^, NO, and H_2_O_2_ are produced by sequence of metal ion-dependent enzymatic reactions that, in turn, may give rise to highly reactive compounds: OH^•^ and hypohalogenous acids, as well as 1O_2_, NO^•^, and NO_2_^•^. During bystander effect, possible participation of the Fenton reaction is evidenced by the studies that showed that the radiation-induced adaptive response depends on the production of the signal molecule NO [11, 131]. Interestingly, in macrophages, the substitution of dG with 8-oxodG in the DNA ligand for TLR9 is accompanied by a significant increase in TNF-*α* cytokine [109]. In other words, an oxidized DNA seems to be a stronger TLR9-stimulating ligand than nonoxidized DNA.

In our opinion, oxidized DNA is one of the components of damage-associated molecular pattern molecules (DAMPs). Its effects can potentially increase when exposure to oxidized DNA is concomitant with the presence of other DAMPs. It might be that effects of oxidized DNA are at least in part mediated by high mobility group box 1 (HMGB1) protein whose expression is enhanced after irradiation. HMGB1 functions as an extracellular damage-associated molecular pattern molecule that promotes inflammation, cellular differentiation, survival, and migration [132–136]. HMGB1 was shown as an essential component of DNA-containing complexes that stimulated cytokine production through a TLR9-MyD88 pathway. Extracellular HMGB1 accelerates the delivery of CpG-DNAs to its receptor, leading to a TLR9-dependent augmentation of IL-6, IL-12, and TNF*α* secretion [137–143]. There is evidence that HMGB1 protein binds preferentially to damaged DNA [144]. It was also shown that extracellular histones directly interact with TLR9 and enhance DNA-mediated TLR9 activation in immune cells [145].

In the populations of irradiated lymphocytes, the expression of *TLR9 *gene and the main adaptor of its signaling pathway MyD88 increase severalfold [27]. In order to confirm participation of TLR9 in bystander DNA-signaling during the development of BE, we blocked these receptors by two inhibitors, a specific oligonucleotide suppressor that provides considerable competition in binding the ligand with the receptor or nonspecific inhibitor chloroquine that changes the pH value in endosome and makes the formation of the DNA-receptor complexes unlikely. When TLR9 pathway was blocked, there were no substantial changes in the localization of 1q12 loci and in the level of NO in bystander cell exposed to ecDNA^R^ from irradiated cells. However, both an increase in the levels of ROS production and an activation of ribosomal genes still took place [23, 30, 34]. These findings suggest that radiation-induced bystander effect may be propagated through more than one molecular pathway. In addition to oxidized DNA-stimulated TLR9 receptors, other sensors, whose activation leads to the changes in ROS and rRNA expression levels but does not lead to transposition of the analyzed chromosomal loci in the nucleus, may be involved. Evidence pointing at existence of toll-like-receptor-independent stress signal transfer pathways was previously demonstrated by other authors [146, 147], including cytoplasmic DNA-dependent STING, AIM2, RIG-1, and DAI sensor pathways [148]. Some of these pathways are directly linked to apoptosis induction by ecDNA fragments (AIM2); others stimulate pro- and anti-inflammatory cytokine synthesis. It is possible that ecDNA^R^ may be uptaken to penetrate into the cytoplasm and activate those pathways. It is also very well might be that eukaryotic cells contain a variety of the molecules that sense the damage in cell-free DNA, and that these cells may differentially respond to a variety of oxidized or otherwise modified DNA bases. The reception of ecDNA produced by irradiated cells warrants further investigations.

After exposure of human lymphocytes to X-ray radiation at a low dose, adaptive reaction develops within 4–6 hours. It is known that such response takes a few cell cycles [149, 150] even longer [125, 151, 152]. Although the persistence of irradiation effects has been described in the literature about 50 years ago, its mechanisms are still to be determined. However, it is very likely that one of the main components of the long-term process of irradiation response is the generation of ROS that may remain elevated for many cellular generations [153, 154]. In our opinion, the oxidative stress may be maintained in cells after the initial irradiation event as a result of sustained activation of oxidized ecDNA-signaling pathway. This activation may be maintained by the fragments of oxidized ecDNA released from irradiated cells that die by apoptosis and release their damaged DNA into circulation or cell media. When the fragments of oxidized ecDNA interact with recipient “bystander” cells, it evokes secondary oxidative stress in some bystander cells. In turn, these bystander cells initiate apoptotic cascades that lead to further release of oxidized ecDNA. The mechanism described above takes into account previous observations that the daughter populations derived from irradiated cells retain an elevated level of ROS that play a substantial role in maintaining the adaptive response throughout cell generations [155].

## 6. Conclusion

Irradiation, chronic diseases, or other prooxidative stimuli and conditions lead to an increase in oxidative stress and in oxidation of cellular DNA. In case of apoptotic death of stressed cells, oxidized DNA ends up released in cell culture medium (ecDNA) or in circulation (cfDNA). In cultured cells, oxidized ecDNA serves as a stress signal that is transmitted from stressed (i.e., irradiated) cells to bystander cells. It is tempting to speculate that a similar process takes place in human body challenged with focused irradiation or suffering from chronic disease. In human cells, oxidized DNA induces additional synthesis of ROS. When ecDNA/cfDNA = dependent increase in ROS levels remains moderate, the bystander cells develop an adaptive response, that is, at least in part, due to an activation of the transcription factor NRF2, which is capable of inducing antioxidant expression program. 

So far, no studies demonstrating that oxidized cfDNA may play a role in bystander effect *in vivo* were published. Effects of exposure to oxidized cfDNA should be taken into account when treating tumors with various ROS-producing agents and irradiation. As oxidized cfDNA released from the dying tumor cells enters the circulation, it is being carried to the distant organs, with its effects expected to be systemic. For example, the damaged DNA released from irradiated cells may be responsible for abscopal effects that are suspected to be depended on actions of immune system, in particular, the ones mediated by TLRs. It is possible that artificial modulation of concentration, GC-content, and the level of oxidation of cfDNA may improve clinical outcomes in patients with various chronic diseases accompanied by extensive cell death. The data summarized above indicate the necessity for further study of the effects of oxidized DNA in both *in vitro *and *in vivo *systems.

## Abbreviations

AR: Adaptive responses
BE: Bystander effect
DSBs: DNA double-strand breaks
EcDNA: Extracellular DNA
OS: Oxidative stress
ROS: Reactive oxygen species
HUVEC: Human umbilical vein endothelial cells
MSC: Mesenchymal stem cells.
